# Detecting Lung Diseases from Exhaled Aerosols: Non-Invasive Lung Diagnosis Using Fractal Analysis and SVM Classification

**DOI:** 10.1371/journal.pone.0139511

**Published:** 2015-09-30

**Authors:** Jinxiang Xi, Weizhong Zhao, Jiayao Eddie Yuan, JongWon Kim, Xiuhua Si, Xiaowei Xu

**Affiliations:** 1 School of Engineering and Technology, Central Michigan University, Mount Pleasant, Michigan, United States of America; 2 College of Information Engineering, Xiangtan University, Xiangtan, Hunan Province, China; 3 Division of Bioinformatics and Biostatistics, National Center for Toxicological Research, Jefferson, Arkansas, United States of America; 4 College of Engineering, University of Georgia, Athens, Georgia, United States of America; 5 Department of Mechanical Engineering, California Baptist University, Riverside, California, United States of America; 6 Department of Information Science, University of Arkansas, Little Rock, Arkansas, United States of America; Technion—Israel Institute of Technology, ISRAEL

## Abstract

**Background:**

Each lung structure exhales a unique pattern of aerosols, which can be used to detect and monitor lung diseases non-invasively. The challenges are accurately interpreting the exhaled aerosol fingerprints and quantitatively correlating them to the lung diseases.

**Objective and Methods:**

In this study, we presented a paradigm of an exhaled aerosol test that addresses the above two challenges and is promising to detect the site and severity of lung diseases. This paradigm consists of two steps: image feature extraction using sub-regional fractal analysis and data classification using a support vector machine (SVM). Numerical experiments were conducted to evaluate the feasibility of the breath test in four asthmatic lung models. A high-fidelity image-CFD approach was employed to compute the exhaled aerosol patterns under different disease conditions.

**Findings:**

By employing the 10-fold cross-validation method, we achieved 100% classification accuracy among four asthmatic models using an ideal 108-sample dataset and 99.1% accuracy using a more realistic 324-sample dataset. The fractal-SVM classifier has been shown to be robust, highly sensitive to structural variations, and inherently suitable for investigating aerosol-disease correlations.

**Conclusion:**

For the first time, this study quantitatively linked the exhaled aerosol patterns with their underlying diseases and set the stage for the development of a computer-aided diagnostic system for non-invasive detection of obstructive respiratory diseases.

## Introduction

The ability to diagnose lung cancer at an early stage is crucial to patients’ survival. Despite extensive research, there is still a severe lack of techniques capable of early cancer detection. Even though diagnostic tools such as chest radiography, computed tomography, and biopsy are accurate in diagnosis, they have not been recommended for screening purposes. The benefits of these tools outweighing their invasive nature and potential risks to the patients have not been substantiated to be extensively used for screening purposes.

Exhaled breath contains clues to many lung diseases, which can be related either to the metabolic changes in cancer cells or lung structure remodeling[1]. Analyzing exhaled breath from individuals who are at a high risk of lung cancer could be an inexpensive and non-invasive method of diagnosing the disease. Breath analysis has been conducted in either gas phase as exhaled breath or liquid phase as exhaled breath condensates (EBCs)[2]. In the first approach, a unique gas “*fingerprint*” is correlated with a particular disease. Examples include increased concentrations of antioxidants for chronic obstructive pulmonary disease (COPD)[3], nitric oxide for asthma[4], and isoprene for non-small cell lung cancer (NSCLC)[5]. However, these *gas-fingerprint* based devices, such as electronic noses[6], only measure the concentration of exhaled gaseous chemicals. They do not provide information regarding where these chemicals are produced (the cancer site) or the level of lung structural remodeling, both of which are essential in treatment planning. In the second approach, non-volatile molecules exhaled from the fluid that lines the lung are collected as condensates. This method has been shown to be useful in studying inflammatory and oxidative processes on the surfaces of the respiratory tract[7]. However, this method is limited by the lack of standardization. Exhaled water vapor causes considerable dilution of the non-volatile biomarkers and accounts for more than 99.99% of the collected EBCs[8]. As a result, collection devices can notably influence the collected biomarker levels and values obtained with different instruments are not directly comparable. Saliva and nasal contamination also add to this problem. More importantly, EBCs are from various parts of the respiratory tract, and there is no way to distinguish the EBC fraction from each part. There also exist a third approach, the aerosol bolus dispersion (ABD)[9,10], which uses aerosols to measure lung functions. However, ABD does not provide any new information of the lung health beyond current pulmonary function tests[10].

A new exhaled aerosol test was recently introduced by Xi et al.[11], which is promising to detect a lung disease, grade the severity, and pinpoint the disease site. The underlying hypothesis of this method is that each lung structure has a signature *aerosol fingerprint* (AFP), in contrast to the *gas finger-prints* discussed previously, and that any alteration to the normal pattern is suggestive of a structural variation inside the lung. The AFP-based breath test will be much like using a personal air sampler. The subject first inhales particles at a prescribed speed and depth. During exhalation, the particles are collected on a mouth-filter, which will be further analyzed to evaluate the lung health conditions.

Questions remain regarding this method. For instance, how does one quantify an AFP pattern and distinguish different AFP patterns accurately? How does one determine the information (presence, site, grade, etc.) of a lung disease from a given AFP sample? Will this method be sensitive to small airway changes? Will this method be robust enough to tolerate test uncertainties? The first question was addressed in Xi et al.[12,13] where multiple morphological measures were exploited to differentiate AFP patterns in a quantitative manner, such as the spatial distribution, lacunarity, fractal dimension, and multifractal spectrum. Fractal dimension (FD) is a measure of the complexity or irregularity of an object’s structure. FD was selected to describe the complex AFP patterns because human lungs are actual space-filling fractal structures[14,15,16] and have an *in vivo* measured FD of 1.57 or so [17]. Therefore, it is expected that exhaled aerosols also exhibit fractal characteristics. In addition, lung cancer growth is accompanied by airway remodeling and structural irregularities, which can be readily described using FDs. Fractal features have been probed in the pathology and grading of cancers in lungs[18], breasts[19], kidneys[20], among others. It provides a simple tool to describe complex systems and allows objective evaluations that do not rely on the experience of the examiner.

**Fig 1:**
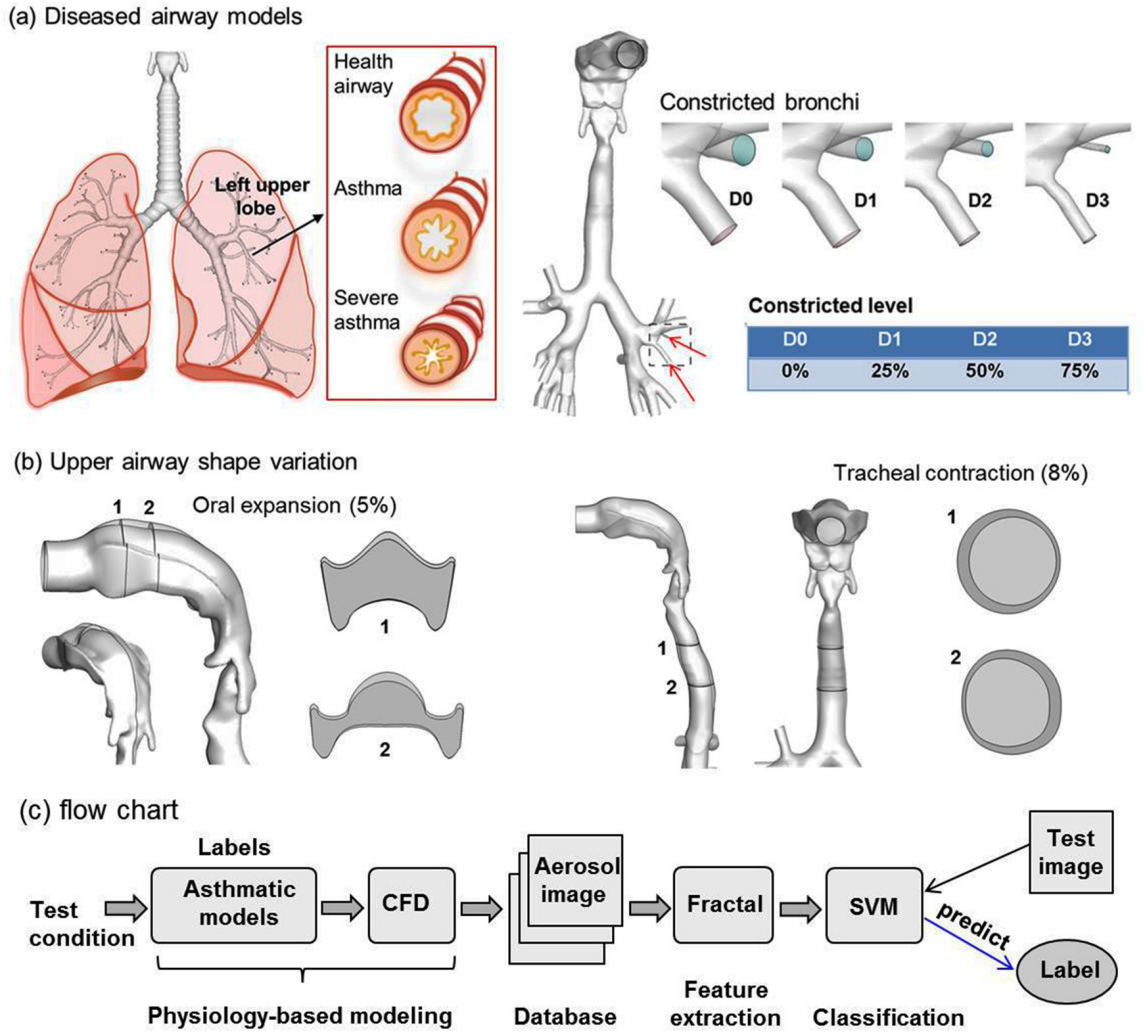
Asthmatic models (a), upper airway variation (b), and flow chart (c) of the proposed method methodology. The airway constriction level in the asthmatic models ranges from 0% (D0) to 75% (D3). The shape variations represent the potential uncertainties in the upper airway during breath tests. There is 5% oral expansion and 8% tracheal contraction relative to the control cases. Physiology-based modeling was undertaken to generate exhaled aerosol images; the images were characterized using fractal analysis to extract salient features; a SVM classifier was trained with extracted feature vectors and tested with extra samples. CFD: computational fluid dynamics; SVM: support vector machines.

This study will aim to address the remaining three questions, namely how to link exhaled aerosol patterns to internal lung diseases? Is the new method accurate? Is it robust? To this aim, we will introduce an image-CFD-fractal-SVM model and evaluate the feasibility of the proposed exhaled aerosol test using this model. This new model includes four steps: sample acquisition, fractal feature extraction, database quality evaluation, and SVM classification (flow chart in Fig 1). The performance of the proposed model will be assessed in four asthmatic models (Fig 1) using two datasets that represent ideal and more realistic testing conditions (Table 1). Specific aims of this study include: (1) database development of aerosol samples to train and test the classification model, (2) fractal feature extraction to quantitatively describe exhaled aerosol images, (3) SVM classification to correlate exhaled aerosol features to the asthmatic grade, and (4) analysis of data quality (before test) and misdiagnosed samples (after test) to minimize misclassifications. This new model will set the stage for developing a non-invasive, computer-aided diagnostic system that is capable of rapid detection and location of asthmatic bronchitis using exhaled aerosol tests.

**Table 1:** Test variables and their ranges.

Experiments	Test variables	Range of variables
**108-sample** (= 3×4×9)	Flow rate (L/min)	27, 30, 33
	Asthmatic model	D0 (normal), D1 (25%), D2 (50%), D3 (75%)
	Particle diameter (μm)	0.2, 0.4, 0.6, 0.8, 1, 2, 3, 4, 5
**324-sample** (= 3×4×9×3)	Flow rate (L/min)	D0, D1, D2, D3
	Asthmatic model	D0, D1, D2, D3
	Particle diameter (μm)	0.2, 0.4, 0.6, 0.8, 1, 2, 3, 4, 5
	Upper airway (UA)	Upper airway (UA)

## Methods

### Study design

A database containing two datasets will be developed using four asthmatic models and nine particle sizes (Table 1). The first dataset contains 108 samples and represents an ideal test environment with small respiration fluctuations (30±10% L/min) and no upper airway variation. The second dataset contains 324 samples and allows for more realistic scenarios such as large fluctuations in both respirations (30±33% L/min) and upper airway geometries (5% oral cavity expansion, and 8% tracheal contraction). To evaluate the uncertainties from potential variations in the upper airway geometry, airway models with oral expansion (5%) and tracheal contraction (8%) were considered relative to the control cases (Fig 1B). The flow chart (Fig 1C) shows the three major steps: physiology-based modeling to generate exhaled aerosol images, image feature using fractal analysis, and SVM classification.

### Physiology-based simulation of the exhaled aerosol test

An image-based mouth-throat airway model developed by Xi and Longest[21] was used to represent the normal airway. Details of the geometry dimensions and modeling procedures were given in Xi and Longest[21,22]. The usage of scan images has been approved by the Virginia Commonwealth University Institutional Review Board. All patient records were de-identified prior to analysis. This model geometry was modified to produce the three asthmatic models by progressively decreasing the diameters of two segmental bronchioles, as illustrated in Fig 1A. The information on location, size, and airway blockage rate of the three asthmatic models is listed in Table 1.

In order to acquire images of the exhaled aerosol patterns, both inhalation and exhalation were simulated, with particles first inhaled and then collected at the mouth exit during exhalation. A blunt profile of the inlet velocity was adopted to approximate the smooth transition from the core flow to the no-slip wall condition[23]. The inlet particle profiles were generated using a stochastic algorithm[24]. For each airway model, five inlet particle profiles were tested for later statistical analysis. In human respiratory flows, both laminar and turbulent flows are expected. The multiple flow regimes were simulated using a large eddy simulation model LES-WALE[25]. A Lagrangian-tracking algorithm was used to simulate the particle motion by integrating the particle transport equation[21],

$$\frac{dv_{i}}{dt}=\;\frac{f}{\tau_{p}C_{c}}\left(u_{i}-v_{i}\right)+g_{i}\left(1-\alpha \right)+f_{i,Brownian}+f_{i,lift}$$ (1)

where *u*_*i*_ is the local fluid velocity, *v*_*i*_ is the particle velocity, *τ*_*p*_ is the particle characteristic time, *f* is the drag factor[26], and *C*_*c*_ is the Cunningham correction factor[27]. User defined functions (UDFs) were developed to account for the effects of near-wall damping[22] and finite particle inertia[28]. The UDF-enhanced Lagrangian-tracking model had provided close agreements with *in vitro* measurements in our previous studies for both submicrometer[28] and micron aerosols[24,29,30]. The computational meshes of the airway models were generated with ANSYS ICEM CFD (Ansys, Inc). A grid sensitivity analysis was conducted by testing the effects of different mesh densities[11,12]. The final mesh consisted of about 2 million elements with a height of 0.05 mm of the first near-wall cell.

### Feature extraction via fractal analysis

In the second step, the numerically predicted aerosols (or AFPs) were visualized in terms of both particle locations and concentration distributions. The resultant images were then quantified using the box-counting fractal dimension analysis. The box-counting fractal dimension (D_B_) measures the complexity of the aerosol pattern and can detect subtle pattern evolutions among asthmatic models, thus allowing effective detection and grading of the disease. The value of D_B_ was calculated as the regression slope of the logarithmic plot between the box size ɛ and the number of boxes N_ɛ_, that contain image pixels[31].

$$D_{B}=\text{ln}\;N_{\varepsilon }\;/\;\text{ln}\;\varepsilon$$ (2)

To extract a feature vector that could adequately represent the aerosol pattern, the image was divided into a 6×6 matrix, with fractal analysis being conducted on each grid. An open source code *ImageJ* with FracLac plugin was used to calculate D_B_[31].

### Disease classification via support vector machine (SVM)

#### Preprocessing procedure

The original dataset consisted of 396 samples with different disease levels (D: D0, D1, D2 and D3), respiration rates (Q: 20, 27, 30, 33, 40 L/min) and upper airway (UA) variations (control, oral expansion, and tracheal contraction). Each sample was presented as 36 continuous-valued feature vectors. Four features with zero-variance were removed (i.e., the four corners of the image). The remaining 32 features were utilized for classification analysis.

To check the quality of the database, principal component analysis (PCA) was implemented to project the feature variables into a new mutually orthogonal space, where the new eigenvectors (PC1, PC2, PC3) were uncorrelated[32]. The advantage of PCA is that it can reduce the number of feature variables of the database without losing major information. This is particularly useful when the scatter of a high-dimensional data set is of interest. PCA is a second order statistical method and only requires information in the covariance matrix of the input data to compute the principal components[32]. One-way analysis of variance (ANOVA) and Tukey’s method with stacked data were used to evaluate the variability of samples and determine the major influential factors. Minitab 17 (State College, PA) was used for PCA transformation and statistical analysis on the image feature database.

#### Classification

A support vector machine (SVM) classifier was selected to correlate the images with diseases based on its high classification accuracy than traditional algorithms, such as decision trees, k-nearest neighbors, and neural networks[33]. SVM aims to obtain a hyperplane separating different data with maximum-margin in a transformed feature space[34]. Support vectors are the data samples closest to the hyperplane. In 1995, Cortes and Vapnik[34] introduced a *soft margin* method that allowed for mislabeled samples and used a soft margin parameter *C* to penalize clustering errors. The optimized classification becomes a tradeoff between a larger margin and a smaller error penalty by simultaneously maximizing the geometric margin and minimizing the classification error. The SVM performance depends on the selection of the kernel and the soft margin parameter *C*. In this study, the Gaussian radial basis function *k*(*x*_*i*_, *x*_*j*_) = *exp*(-γ||*x*_*i*_−*x*_*j*_||^2^) was selected as the kernel function, where *x*_*i*_ is the feature vector and γ is the Gaussian kernel parameter[34]. The soft margin parameter C and kernel parameter γ was selected as 100 and 1/32, respectively. It is noted that SVM is inherently a two-class classifier. A multi-class SVM classifier can be developed by comparing every pair of classes. Classification is achieved by a max-wins voting strategy, where each two-class classifier assigns the test sample to one class; the class with the most votes determines the test sample’s classification[35].

In this study, a multi-class SVM algorithm and 10-fold cross-validation were used for the classification analysis. In the 10-fold cross-validation, the dataset was randomly divided into 10 subsets and the SVM algorithm was run 10 times. In each run, 9 subsets were used as a training set to obtain a SVM classifier, and the remaining one subset was used as a testing set of the obtained SVM. The number of misclassified samples was recorded for each run. After 10 runs, each subset was tested once and trained 9 times. The final accuracy was calculated as follows:

$$Accuracy=1-\frac{total\;number\;of\;misclassified\;samples}{total\;number\;of\;samples}$$ (3)

The function “*svm*” in *R* package “*e1071*” was utilized to train and test the classifier.

#### Distance matrix

Distance matrix analysis was also performed on the dataset. The selected 32 features were used to represent samples. The dissimilarities between samples were measured by Euclidean distance and the values ranged from 0 to 1. The samples were subsequently rearranged through the hierarchical clustering based on dissimilarities between samples. The results of the hierarchical clustering were presented in a heat-map graph.

## Results

### Airflow field

**Fig 2:**
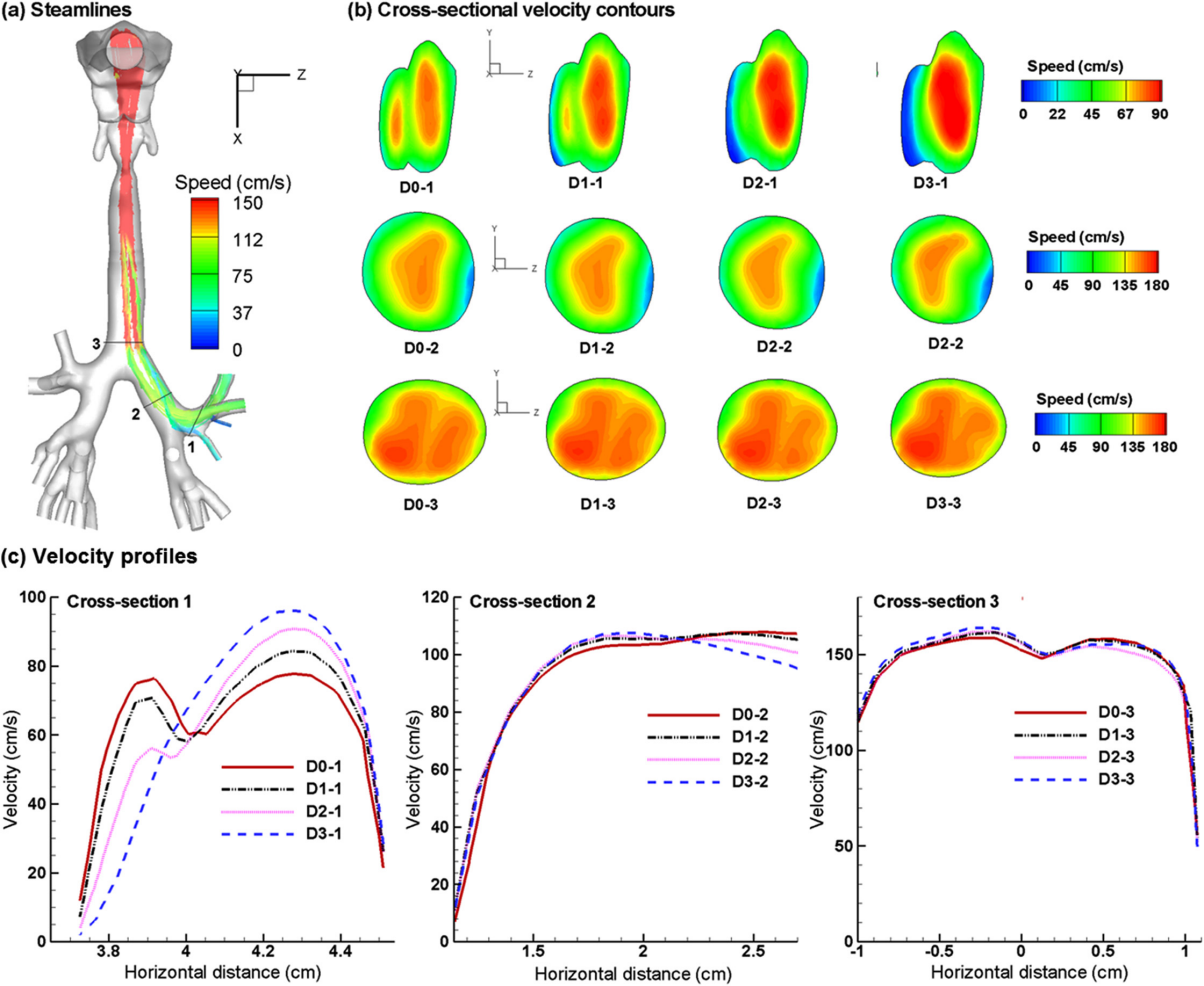
Numerically predicted expiratory flows for the four asthmatic models with varying airway constriction levels. (a) Streamlines, (b) cross-sectional velocity contours, and (c) horizontal velocity profiles.

The predicted expiratory airflows among the four asthmatic cases are compared in Fig 2 in the form of streamlines, cross-sectional contours, and 2-D velocity plots. The asthmatic airway constriction noticeably distorts the streamlines and airflow field (Fig 2A and 2B). In contrast to the two peaks in the velocity contour at Slice 1–1’ for the normal case (D0-1 in Fig 2B), one of the peaks diminishes with increasing constriction level and becomes invisible for D2 and D3. At the same time, a low-speed zone forms near the bifurcation ridge that is next to the constricted bronchioles, which is most obvious in D3 (D3-1 in Fig 2B). These flow disturbances will be conveyed further downstream by the expiratory flow. Due to reduced flow areas from asthmatic constrictions, higher flow resistances are expected; under the same breathing efforts, the respiratory flow rate will be lower. The airway loss prevents aerosols from being inhaled and exhaled smoothly and is expected to noticeably alter the exhaled aerosol profiles. Fig 2C shows the velocity profiles at Slice 1–1’ among the four models in two different directions (a-a’ and b-b’). As expected, flow velocity progressively decreases as the airway constriction level increases. The differences in airflows diminish progressively towards the mouth. It is noted that particle profiles depend on both local flows and particle histories. Although the downstream airflows may appear similar, the particle profiles can still be different because of their time-integrative natures.

### Patterns and fractal feature extraction of exhaled particles

**Fig 3:**
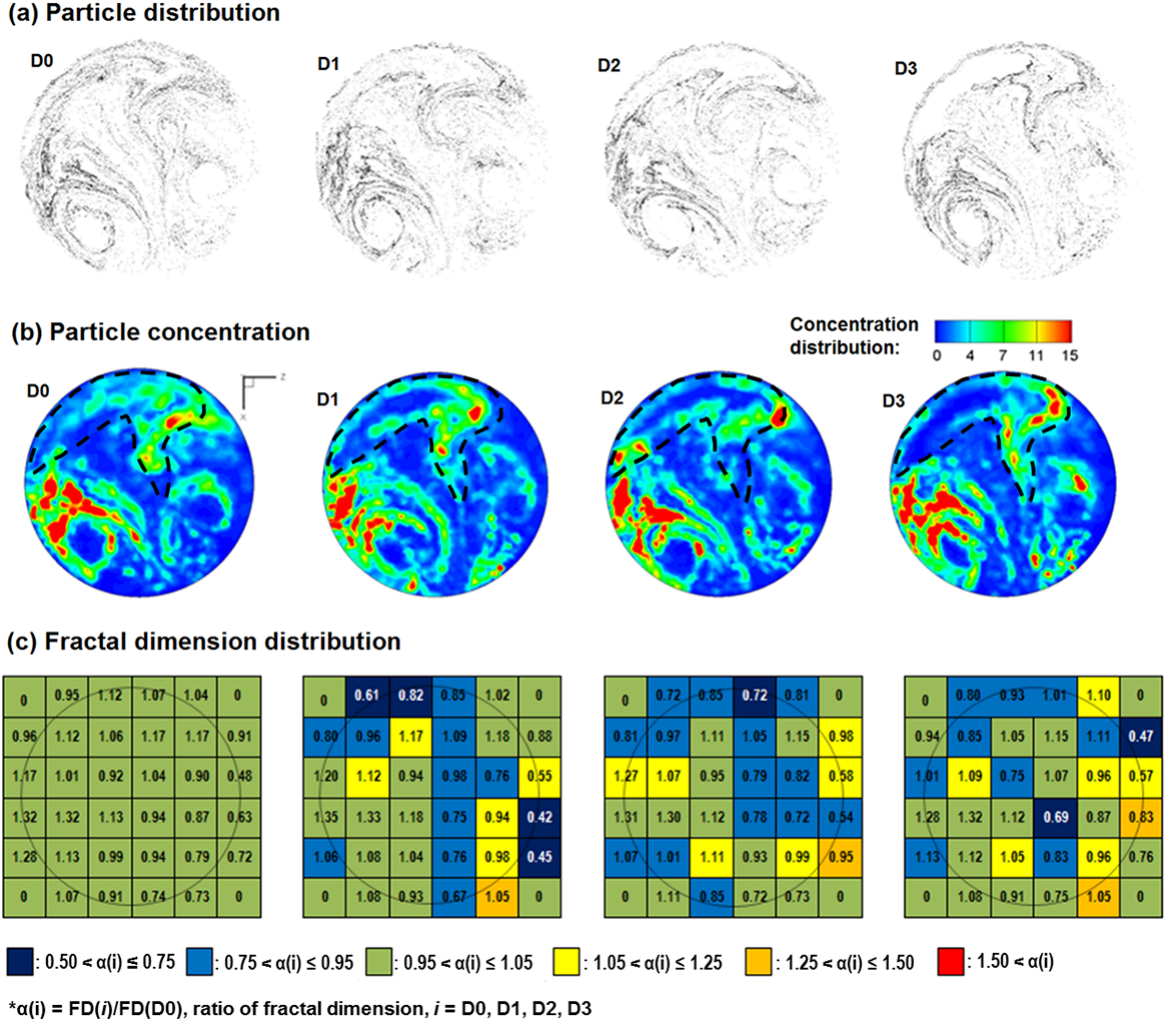
Comparison of exhaled aerosol fingerprints (AFPs) among asthmatic models with varying constriction levels. (a) Particle distribution, (b) particle concentration distribution, and (c) fractal dimension distribution in a 6×6 matrix. The region of interest in (b) is outlined by the dashed contour and represents the most pronounced variations in exhaled aerosol patterns among the four models.

Exhaled particles collect into a unique pattern on the filter and can be seen as the “fingerprint” of the lung. The exhaled particle patterns of the four asthmatic models are illustrated in Fig 3A for 1 μm particles at 30 L/min. Both similarities and disparities in the particle distribution patterns were observed among models, the latter of which was presumably caused by the increasing level of airway constrictions. There were increasing particle attenuations in the upper image, which eventually grew into a crescent-shaped region depleted of particles. This observation was reasonable as there was a gradual airway loss in the four asthmatic models. The amount of particles exhaled from the constricted bronchioles also decreased gradually from D0 to D3. In the extremely constricted scenario (D3, 80% constriction), very few particles could be exhaled. Therefore, the particle-attenuation region is directly related to the disease site and can be selected as the region of interest (ROI) for later analysis. In light of the similarities, two vortices were apparent in the left lower and right lower areas. These two vortices were asymmetric along the central line of the circle, which might result from the right-left lung asymmetry. Considering the possibilities of particle overlapping that prevents an accurate visual interpretation of particle distributions, particle concentrations are also calculated, as shown in Fig 3B. Here blue represents zero concentration and red represents the maximum concentration. Two particle hot spots are apparent in Fig 3B, with one above the left vortex and the other at the right upper corner bordering the crescent-shaped particle-depletion region.

To further characterize these APF images, each image was divided into a 6×6 grid and the fractal dimension on each sub-region was calculated (Fig 3C). In each sub-region, the color is based on the ratio α(i) = FD(*i*)/FD(D0), *i* = D0, D1, D2, D3, with dark blue representing the lowest and red the highest. Again, the 6×6 color arrays are unique to each asthmatic model. Each sample was described using a 36-dimensional feature vector, with each feature being the FD in one sub-region. Four features with zero-variance at the four corners of the image were discarded and the remaining 32 features were used for classification analysis.

**Fig 4:**
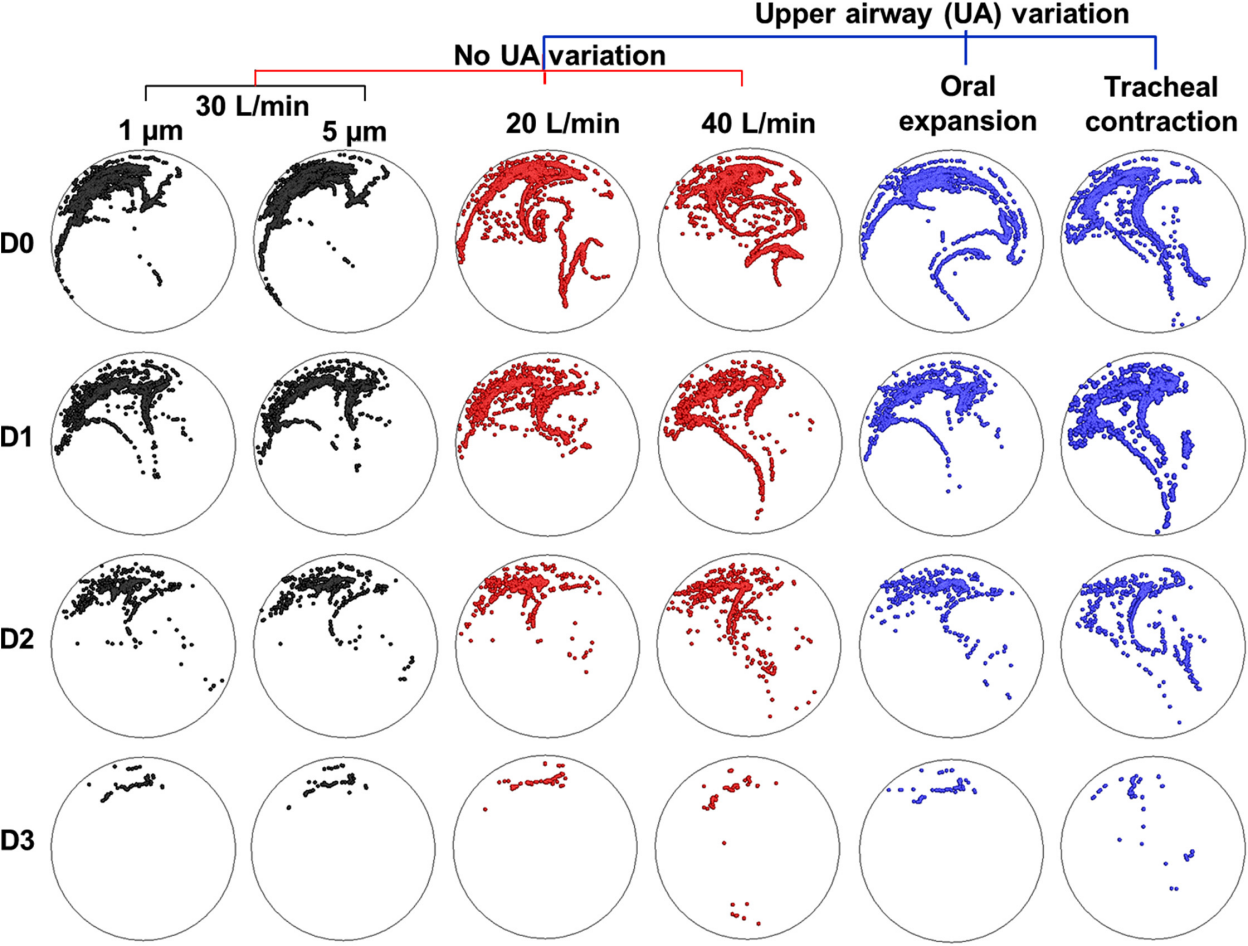
Comparison of exhaled distributions of the particles released from the diseased sites among conditions with different particle sizes, inhalation flow rates, and upper airway geometries. Each column represents a comparison among the four asthmatic models for a given test condition. Each color comprises two columns and represents a comparison by varying one test parameter. Both similarities and differences were observed among the cases.

Fig 4 presents examples of exhaled profiles of the particles released only from the constricted bronchioles. For comparison purposes, images were arranged in a matrix format so that the evolution of particle profiles for a given test parameter could be shown, while other test parameters were fixed. Each column represents a comparison among the four asthmatic models for a given test condition. Each color is comprised of two columns and represents a comparison between two variants of one test parameter, such as particle size, breathing condition, and upper airway geometry. Both similarities and differences were observed among the cases. In particular, dramatic differences exist among D0–D3 for all test conditions, with a progressive particle loss from D0 to D3. Furthermore, exhaled aerosols are very complex and irregular in pattern. Some of them are not readily distinguishable. Because of this, it is a significant challenge to quantitatively characterize the images and correlate these exterior images to the interior lung diseases. An automated technique is also needed to quickly quantify the images that generally have an extensive database.

### Data quality evaluation

**Fig 5:**
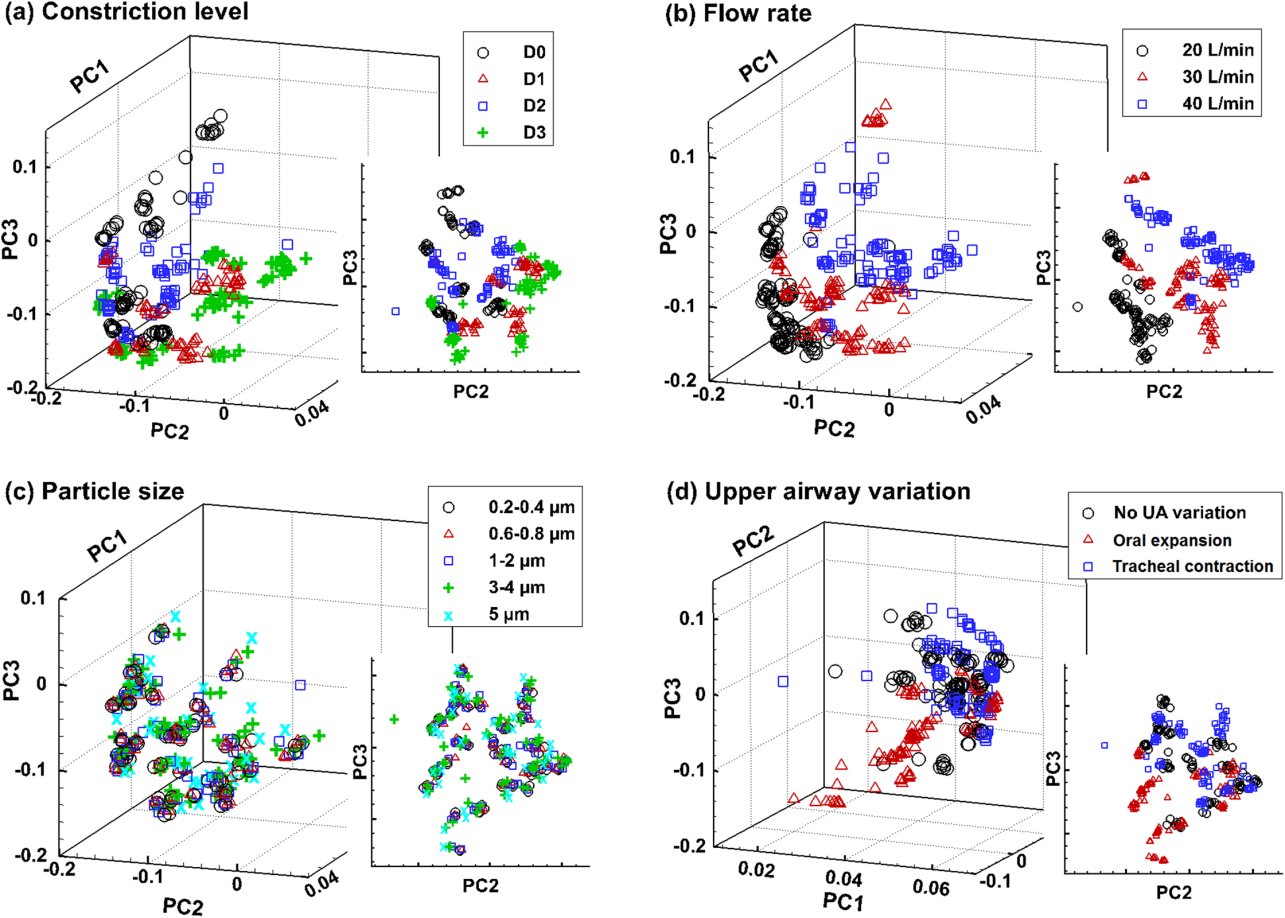
Principle component analysis (PCA) of a database containing 324 sample vectors in a 36-dimensional space. Each vector was transformed via PCA into three mutually orthogonal principle components, which was plotted according to different categories: (a) bronchial constriction level, (b) inhalation flow rate, (c) particle size, and (d) upper airway variation. Varying degrees of data clustering exist among the four categories, with the particle sizes showing nearly no separation.

There were two datasets generated via physiological modeling. The first dataset contained 108 samples and represented ideal test conditions with small respiration fluctuations (±10%) and no upper airway variation. The second dataset contained 324 samples and allowed for more realistic scenarios such as large variations in both respirations (±33%) and upper airway geometries (5% oral cavity expansion, and 8% tracheal contraction). Principal component analysis (PCA) was performed to assess the quality of the 324-sample dataset. PCA projected the feature variables (36 in this study) into three mutually orthogonal eigenvectors (PC1, PC2, and PC3), and thus reduced the number of feature variables for better visual inspection (Fig 5). In other words, PCA reduced 36 dimensions into 3 dimensions that better present the data variance. Varying degrees of clustering were noted among the four categories. The most apparent data separation was observed in flow rate (Q = 20, 30, 40 L/min), followed by the airway constriction level (D0–3) and upper airway variation (Fig 5A, 5B and 5D). Considering the effects of upper airway variations, oral expansions were found to separate themselves more clearly from the control cases than tracheal contractions (Fig 5D). No clear separation was noted when varying the particle size (Fig 5C).

**Fig 6:**
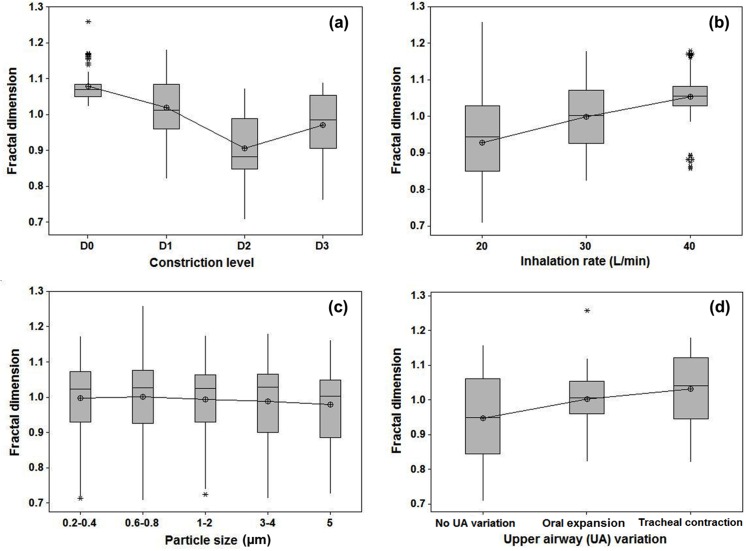
Box plots of the fractal dimension distributions in the region of interest (ROI) with respect to different categories. (a) Bronchial constriction level, (b) inhalation flow rate, (c) particle size, and (d) upper airway variation. The selected ROI is the fourth cell in the 6×6 grid in Fig 3.

As opposed to assessing the overall data quality via PCA, the 324-sample dataset was also evaluated in selected regions of interest (ROI) using the analysis of variance (ANOVA). Fig 6 shows the box plots of the local fractal dimension (FD) distributions as a function of different categories. The selected ROI is the fourth cell in the 6×6 grid in Fig 3. Statistically significantly lower FDs were found in the three asthmatic cases (D1–3) than the FDs for the non-asthmatic controls (D0), indicating a decrease in space-filling ability (Fig 6A). Similarly, there were significant variations in FD for both the inhalation flow rate and upper airway geometry (Fig 6B and 6D). No significant difference in FD was found for the particle size in the range of 0.2−5.0 μm that was considered in this study (Fig 6C).

### SVM classification

Two numerical experiments were conducted to evaluate the performance of the proposed exhaled aerosol test methodology. The first experiment utilized the 108-sample dataset, which has a respiration range of 30±3 L/min with no upper airway variation. In order to compare the influences between the local airway loss and the breathing variation, the samples were labeled with both D-features (D0, D1, D2 and D3) and Q-features (Q27, Q30 and Q33). Each sample was represented as a 32-dimenionsal feature vector. SVM classification analysis was conducted using 10-fold cross-validation (CV), and included two parts. The first part evaluated the dataset with respect to the asthmatic level (D-feature) while the second part to the flow rate (Q-feature). In both analyses, the classification accuracy was 100% (Table 2), showing that both D- and Q-features affect the sample patterns.

**Table 2:** The number of misclassified samples for each run and the final accuracy using 10-fold cross-validation.

	Run	1	2	3	4	5	6	7	8	9	10	Total	Accuracy
**108-sample (D-feature)**	Training	0	0	0	0	0	0	0	0	0	0	0	100%
	Testing	0	0	0	0	0	0	0	0	0	0	0	100%
**108-sample (Q-feature)**	Training	0	0	0	0	0	0	0	0	0	0	0	100%
	Testing	0	0	0	0	0	0	0	0	0	0	0	100%
**324-sample (10-fold CV 1)**	Training	0	0	0	0	0	0	0	0	0	0	0	100%
	Testing	0	0	1	0	0	2	0	0	0	0	3	99.1%
**324-sample (10-fold CV 2)**	Training	0	0	0	0	0	0	0	0	0	0	0	100%
	Testing	1	0	0	1	0	0	0	1	0	0	3	99.1%

**Fig 7:**
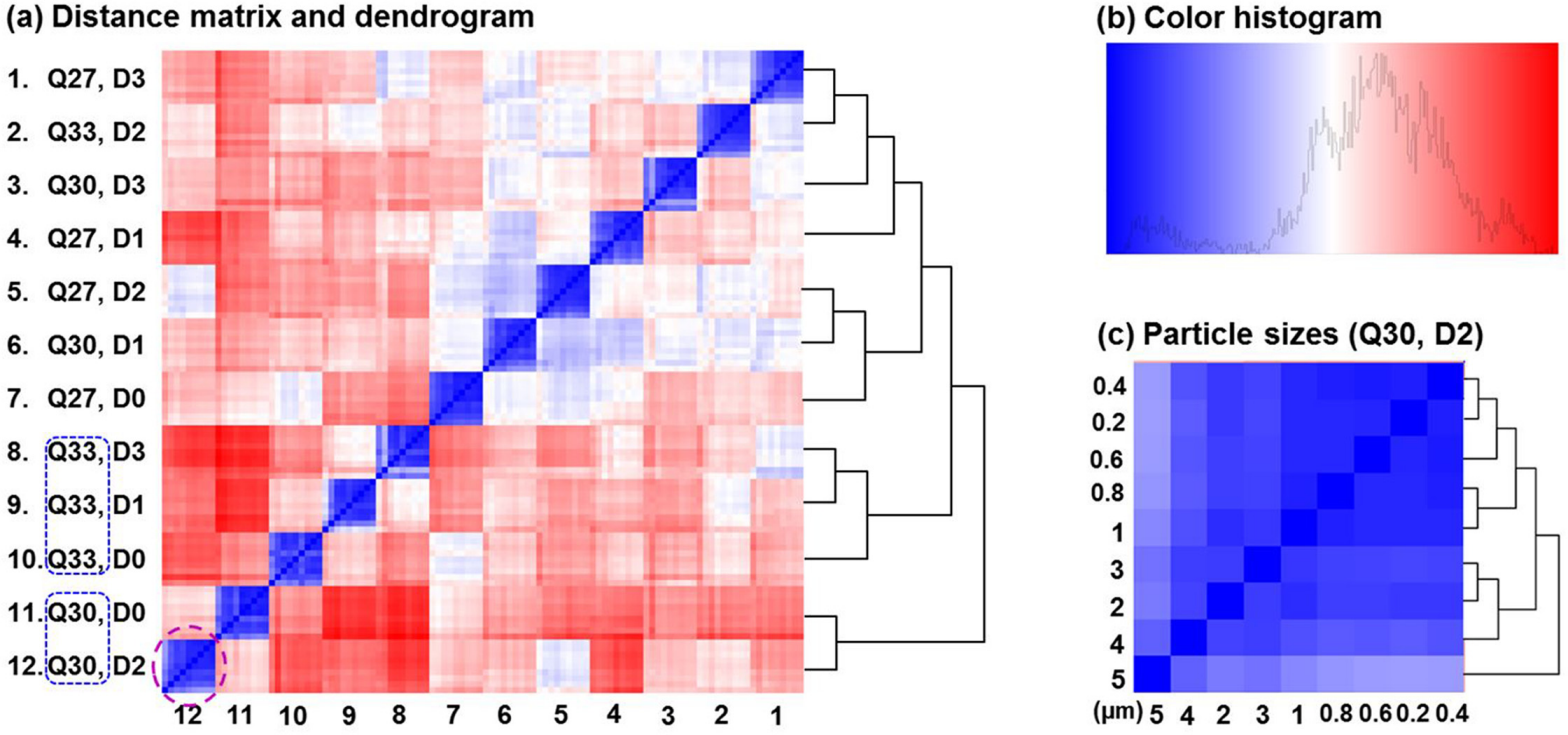
Distance matrix and dendrogram for the 108-sample-experiment which has a respiration range of 30±3 L/min with no upper airway variation. Detailed dendrograms showing hierarchical clustering of particle sizes have been cut off for visual clarity in (a). The color histogram is shown in (b) and an example of the particle dendrogram at the location ([12, 12]: Q30, D2) is shown in (c). The prediction accuracy is 100% in this idealized condition. Q30: inhalation flow rate = 30L/min.

The distance matrix for the 108-sample dataset is shown in Fig 7A, with the main feature dendrogram tree shown on the right side. Detailed dendrograms showing hierarchical clustering for particle sizes have been cut off for visual clarity. The color histogram is shown in Fig 7B and an example of the particle dendrogram at the location ([12, 12]: Q30, D2) is shown in Fig 7C. From Fig 7A, the 108 samples were divided into 12 subgroups; each subgroup shares a given combination of D- and Q-features, as displayed on the left side of the heat-map. As a result, the selected 32-dimenisonal feature vectors were adequate in capturing the differences between the D- and Q-feature samples. On the other hand, it was also observed in Fig 7A that the distributions of D- or Q-feature subgroups were mixed in the heat-map. This mixed distribution indicated that in the original space (32-dimensional space), samples with the same D- or Q-feature could not be readily distinguished. Rather, a new transformed space was needed to classify these specimens. Our classification analysis results showed that by mapping samples into high dimensional space, the SVM algorithm could accurately distinguish the samples with different D- or Q-features. The feature classification accuracies were 100% (10-fold cross-validation) in this 108-sample dataset (Table 2).

In the second experiment (324 samples), test conditions were loosened up to include further uncertainties in breathing (30±10 L/min) and upper airway geometry (5% oral cavity expansion, and 8% upper tracheal contraction). In this experiment, only the D-feature (i.e., D1, D2, D3, and D4) was used as the sample label to evaluate the SVM performance in the four-constriction-level classification. The classification results using 10-fold cross-validation are listed in Table 2. A total prediction accuracy of 99.1% was achieved, with only three misdiagnosed samples. To be comparable with other studies, the sensitivities and specificities were also calculated in this experiment. The two-class control-asthma classification sensitivity was 99.6% (1 false negative or Type II error out of 243 positives), and the two-class specificity was 100% (no false positive or Type I error). The four-level classification sensitivity was 100% for D0, and was 98.8% for D1, D2, and D3, with each level (81 samples) having one misclassified sample.

### Analysis of misclassified samples

**Fig 8:**
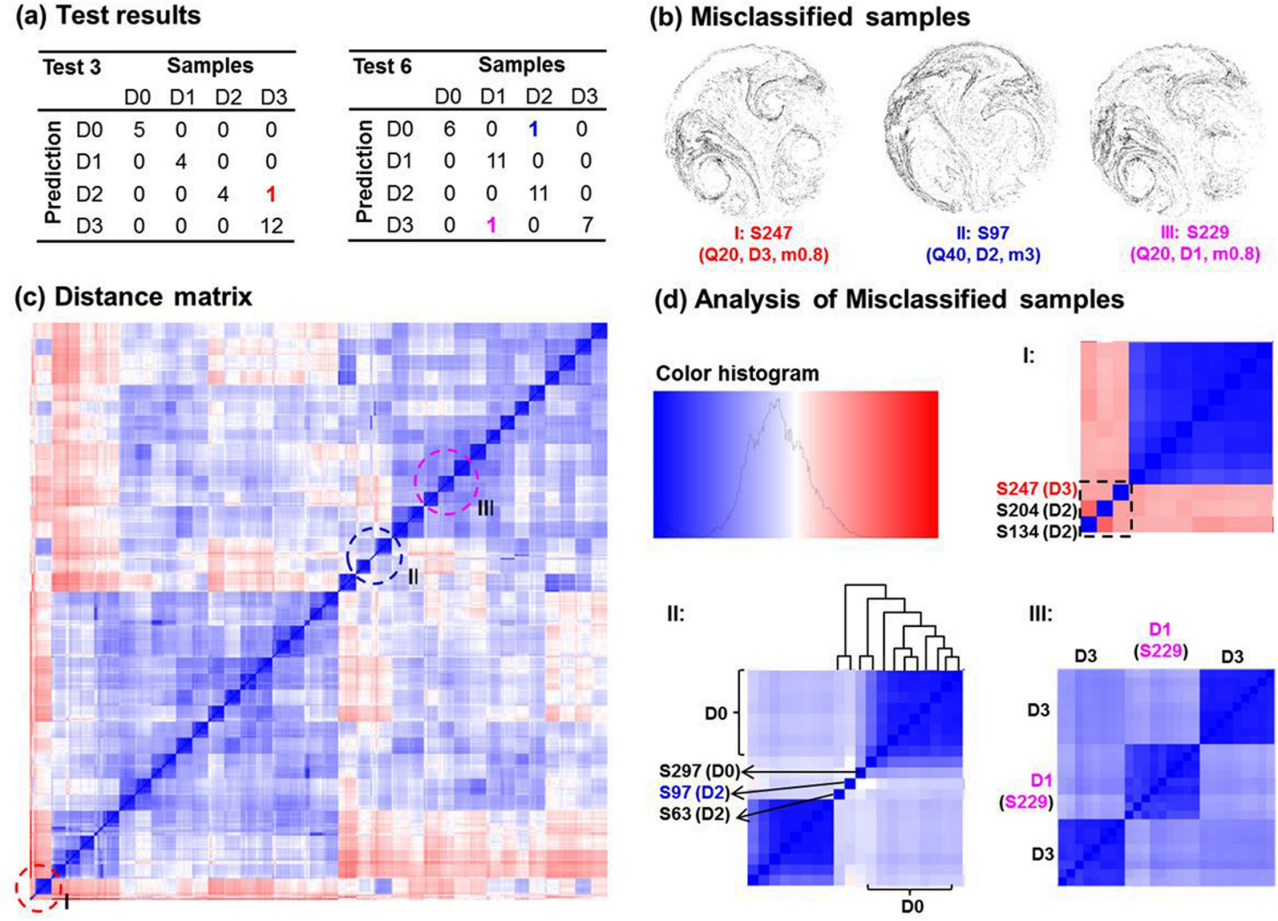
Analysis of misclassified samples in the 324-sample-experiment which has a respiration range of 30±10 L/min and two upper airway variations. The prediction accuracy is 99.1% with three misclassified samples in total (a). The image, sample number, and test parameters of the misclassified samples are listed in (b). The locations of the three misclassified samples are marked in the distance matrix (c), and their zoom-in plots are shown in (d).

We detected three misclassified samples in the 10-fold cross-validation with 324 samples (Fig 8A): S247 (a D3-feature sample being predicted as D2: D3 → D2), S97 (D2 → D0), S229 (D1 → D3). The images and test parameters are shown in Fig 8B. In order to discover the causes of these misclassifications, a detailed analysis was conducted on the above three samples. Fig 8C shows the locations of these three samples in the distance matrix and Fig 8D shows their zoom-in plots. Considering case I (S247), there were three outliers in the lower left corner, one of them being S247 (the third from the right). The other two were S134 and S204, both of which had a D2-feature. Due to large dissimilarities from all other samples, these three samples were clustered into one subgroup, which led to the misclassification of the S247 (D3) as D2. In light of case II (S97), the misclassification of the D2-feature sample as D0 was explained by the dendrogram in Fig 8D. This sample bordered between the D0 and D2 samples, but showed more affinity with the D0 sub-group.

The reason for the third example (case III: D1-labeled S229 being misdiagnosed as D3) was intricate. This time S229 was correctly amalgamated into a D1-subgroup; however, its two neighboring subgroups were both D3-labeled. This might partially explain the misclassification of S229 (D1) as D3. However, another question arose: Why haven’t other samples in the same D1-sub-group as S229 been misclassified? Noticing the secondary similarities (light blue color) among these three subgroups in Fig 8D case III, we speculated that samples in the D1-subgroup could also possibly be misclassified as D3. However, this possibility had been minimized by the selected SVM algorithm, which weighed more toward akin-sample similarities and penalized the influences of apparent outliers. As discussed in Methods, this was achieved by including a Gaussian kernel and a soft margin parameter *C* to maximize the geometric margin and therefore minimize the classification errors.

To test the robustness of the classifier, the 10-fold cross-validation test was repeated by rearranging the 324-dataset into 10 random groups. The same prediction accuracy was obtained (99.1%, with three misclassifications in total). In particular, the same three misclassified samples were identified (this time, S229: D1 → D3 in Test 1, S247: D3 → D2 in Test4, and S97: D2 → D0 in Test 8). This performance consistency instills confidence that (1) the developed classifier performs consistently, and (2) the classifier is sufficient to identify all true outliers (the above three samples in this study).

## Discussion

In this study, a paradigm of an exhaled aerosol test for lung diagnosis was presented and its accuracy was demonstrated. We evaluated its performance using two datasets generated via physiologically-based modeling in four asthmatic lung models. The diagnosis accuracy was 100% under an ideal test condition and 99.1% under more realistic test conditions. Even though the test samples are limited in number, the proposed aerosol breath test shows promising results to be a new tool for screening and diagnosing lung diseases.

The novelty of the aerosol breath test can be established by three aspects. First, compared to current diagnostic tools such as chest radiography, CT and biopsy, this method is non-invasive and easy to perform; therefore, it is well suited for use in screening purposes for subjects with a high level of occupational exposure such as coal mine workers. Second, compared to other breath test techniques in development, such as electronic noses, exhaled breath condensate, and aerosol bolus dispersion, this new method has the unique potential to locate the disease site, which is highly desirable for site-specific drug delivery. Finally, compared to standard classification studies that require images of cancerous tissue samples, this method only requires an image of exhaled aerosol pattern, which can be obtained easily.

Our conventional understanding of the fractal lung is that FD decreases in asthmatic lungs[18]. However, this study reported a more complex correlation between the aerosol FD and asthmatic constriction level. With increasing constriction levels, the aerosol FDs initially decreased and then started to increase for severely constricted conditions (Fig 6A). This finding might appear counter-intuitive, considering that lung FD declined with aggravating asthma and that the FD of exhaled aerosol patterns was expected to decrease in a similar way. This seeming contradiction can be reconciled by considering both the local and global lung features that interactively determine the exhaled aerosol profiles. As asthma exacerbates, the surface irregularity, as well as the structural complexity, at the disease site progressively increases, while the ventilation capacity of the airway tree continuously decreases. The FD increase in D3 (75% constriction) (Fig 6A) may reflect these two competing factors where the local effect becomes predominant. A similar argument has been made in Boser et al.[36], who observed a monotonic decrease in FD in asthmatic lungs but speculated a positive contribution to the overall FD variation if the local surface features were considered. They suggested that an overall FD might not provide a complete description of the diseased airways and recommended additional measures (e.g., local features) be adopted[36]. Indeed, including both the local and global features of the histological images has been shown to remarkably improve the diagnosis of prostate cancers[37]. The saddle point observed in the ROI-based FD in Fig 6A may correctly reflect the two competing factors of global loss in ventilation units *vs*. local increase in surface irregularities. In this study, a 6×6 matrix of sub-regional FDs was implemented to describe the exhaled aerosol features. Considering that asthmatic constrictions may vary vastly in size and location, a multi-resolution matrix scheme can be used for improved search accuracy. Images will be quantified at different scales so that the asthma-associated airway abnormity can be detected with one of these scales.

Particle size in the range of 0.2−5.0 μm was found to have little influence on exhaled aerosol patterns, as shown in Figs 5C and 6C. This insignificant influence may be attributed to the specific physical properties of particles in this range that neither inertia impaction nor molecular diffusion is predominant in particle transport and deposition. This finding is desirable in that polydispersed aerosols around 1.0 μm, which is much easier to generate than monodispersed aerosols, can be used as diagnostic agents without noticeably sacrificing diagnosis outcomes. However, this doesn’t mean that particles of any size are suitable as the diagnostic agents. Large inertial particles (10 μm) was found to cause remarkable differences in exhaled aerosol patterns compared to small particles (≤ 5 μm) as considered in this study [11] and therefore, was not appropriate for this application.

The fractal-SVM classifier developed in this study has been shown to be highly sensitive to geometric variations. Through the exhaled images, it can detect not only major changes in respiratory activities, but also subtle modifications that may not be visually detectable. Employing two databases of computer-generated exhaled aerosol samples and the 10-fold cross-validation method, we achieved 100% accuracy in the 4-grade (D0−3) classification with the ideal 108-sample dataset and 99.1% accuracy with the more realistic 324-sample dataset. It is acknowledged that the high accuracy in this study is presumably attributed to the limited number of disease models (four) considered herein and should not be interpreted as evidence that the proposed breath test outperforms current diagnostic techniques. However, this high prediction accuracy does show the promise of the aerosol breath test to be a sensitive lung diagnostic tool. Considering the complexity of the lung pathology, a large database is needed to include lung diseases of different types, with different grades, and at various locations.

Even with limited sample sizes and relatively ideal test conditions, chances of misdiagnosis still remain. Three misclassified samples were detected in the 324-sample test; their error sources were individually examined using the distance matrix. Specifically, the same three samples were identified in repeated 10-fold cross-validation tests. This consistency indicates a robust performance of the fractal-SVM classifier, which can be a powerful tool for outlier detection. Knowing the origins leading to misclassification is crucial in developing reliable classifiers. Only by minimizing the sources of errors can the classifier be optimized for reliable diagnosis. It is noted that the robustness of the proposed technique was only tested on one asthmatic lung with four constriction levels. Further robustness analyses are needed by testing the technique on more types of lung diseases and a large cohort of patients.

It is noted that the lung disease-aerosol correlation cannot be established in the original feature vector space. A statistical clustering algorithm such as SVM is essential to amalgamate similar samples and separate dissimilar ones. As evident in the two distance matrices in Figs 7 and 8, not all D-feature subgroups have been clustered into one region. Rather, their distribution along the diagonal is somewhat mixed with the Q-feature subgroups, indicating significant influences on classification from both lung structure and respiration flow rate. However, the results demonstrate that the SVM algorithm can distinguish the four-class data with a high accuracy. The fractal-SVM classifier has exhibited a satisfactory robustness. Despite the existence of multiple outliers in the database, SVM is still able to distinguish >99.1% samples in a 324-sample dataset.

An inherent limitation of the proposed diagnostic method is that it relies on image comparisons to diagnose lung diseases and needs a baseline aerosol “fingerprint” of the patient to start with, which the patient may not necessarily have at his/her first visit. This challenge can be partially addressed using a two-step pre-diagnosis, as follows. First, the range of fractals that represents the normal lungs can be obtained from population statistics, which can be further used to determine whether an airway anomaly exit or not. Second, a database containing the image fractals of known obstructive lung diseases can be developed as a reference in hope to preliminarily diagnose the type of the disease. Once the first exhaled aerosol image was recorded, images subsequently acquired can be used to validate the original diagnosis or to monitor the progression of the disease. Moreover, the database development is an on-going process. As more data are collected, the accuracy for both pre-diagnosis at the initial clinic visit and disease monitoring during the treatment could be improved.

Other limitations that may compromise the physiological realism of this study include the assumption of steady flows, rigid airway walls, a constant glottal aperture, a limited number of disease models, and numerical modeling only. Tidal breathing, compliant walls, and a dynamic glottis all will alter the exhaled aerosol patterns and act as compounding factors that lower the diagnosis accuracy at varying degrees. In practice, a standardized aerosol breath test should be established regarding the breath and sitting position to minimize the impacts from such compounding factors. Moreover, the algorithm of this proposed method is based on the differences among images, rather than the image itself. These compounding factors will affect the patterns of the exhaled aerosol images, but will not significantly affect the level of difference among these images. In this sense, assuming steady flows and rigid airways has greatly simplified the modeling process while still capturing the essence of the involved physics. Further studies with more physiologically accurate boundary conditions are desirable to quantify their respective impacts on the prediction accuracy. Concerning the limited number of disease models, future development of a database categorizing more diseases and their respective fractal patterns is necessary. Complementary *in vitro* and *in vivo* tests are needed to validate the numerical predictions in this study.

In summary, the performance of the proposed aerosol breath test was numerically assessed in four asthmatic lung models. The exhaled aerosol patterns were generated using a high-fidelity image-CFD approach and were statistically correlated to their underlying lung diseases using a fractal-SVM model. Results of this study provide the proof-of-concept in developing a computer-aided diagnostic system for rapid detection and localization of asthmatic bronchitis in a non-invasive nature. This diagnostic system can also potentially be used in lung cancer patients.

## Supporting Information

S1 Table Data set for 108 samples. This folder contains tables of Image fractals of 108 samples, the training and test sub-datasets, and the 10-fold cross-validation test results.
(RAR)

S2 Table Data set for 324 samples. This folder contains tables of Image fractals of 324 samples, the training and test sub-datasets, and the 10-fold cross-validation test results.
(RAR)
